# Supplementary figures and images for: Impact of COVID-19 lockdown on psychosocial factors, health, and lifestyle in Scottish octogenarians: The Lothian Birth Cohort 1936 study
Source: PLoS One. 2021 Jun 17;16(6):e0253153. doi: 10.1371/journal.pone.0253153 (PMC8211159; doi:10.1371/journal.pone.0253153)

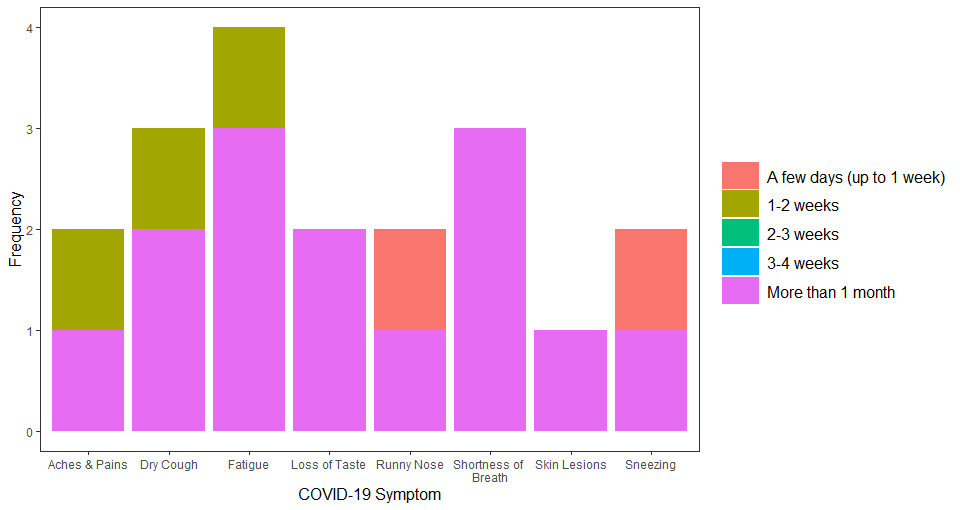

Supplement: S1 Fig — (DOCX) [file pone.0253153.s002.docx]

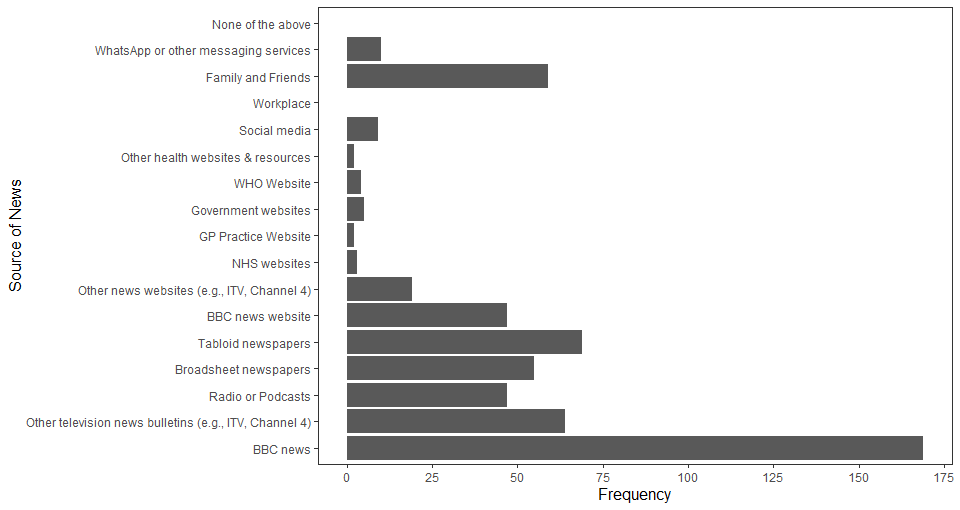

Supplement: S2 Fig — (DOCX) [file pone.0253153.s003.docx]

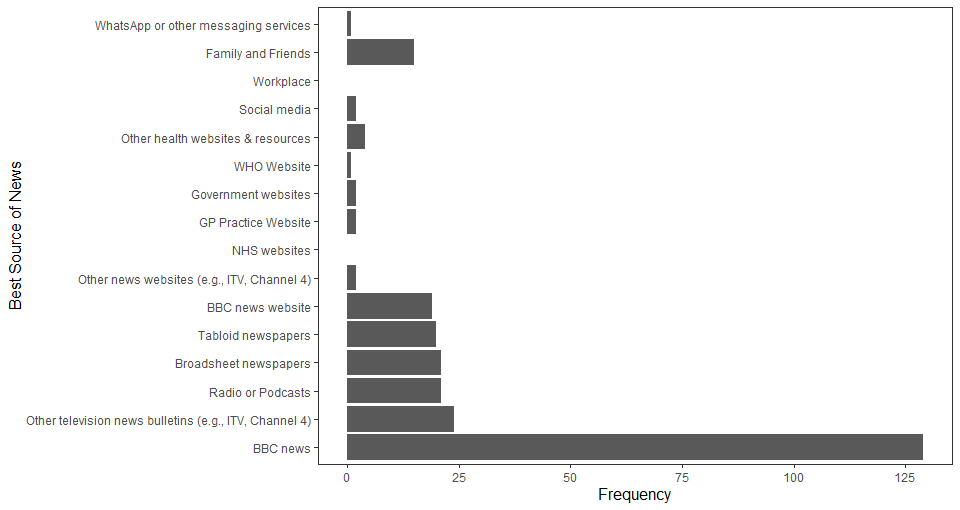

Supplement: S3 Fig — (DOCX) [file pone.0253153.s004.docx]

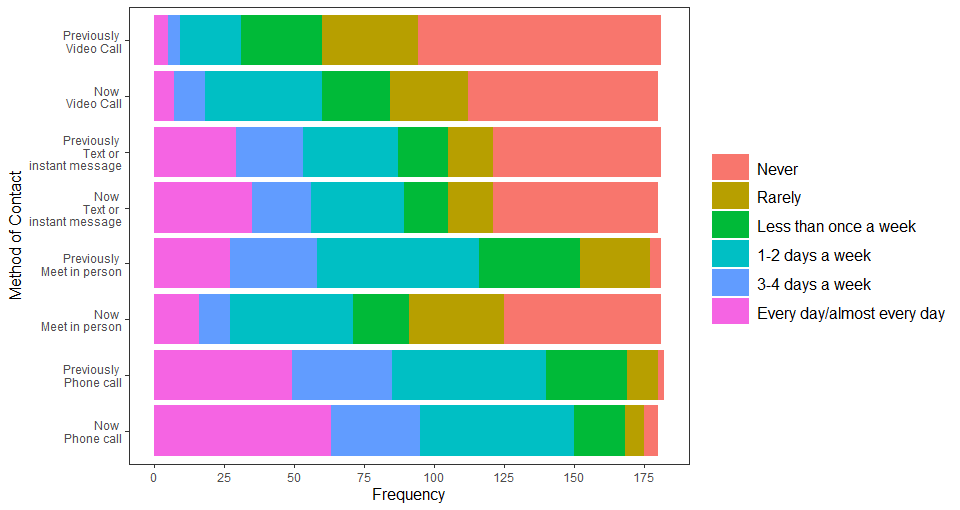

Supplement: S4 Fig — (DOCX) [file pone.0253153.s005.docx]

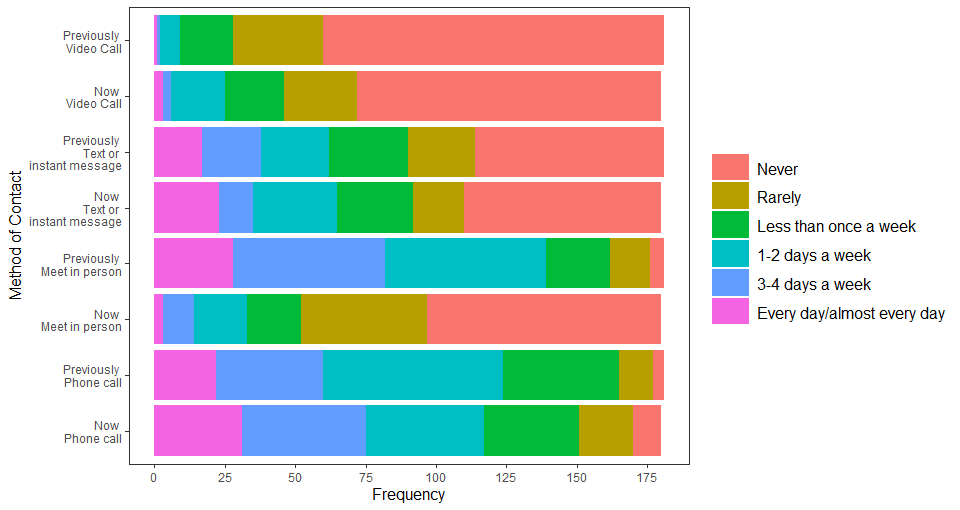

Supplement: S5 Fig — (DOCX) [file pone.0253153.s006.docx]
