# Supplementary material for: Impact of COVID-19 lockdown on psychosocial factors, health, and lifestyle in Scottish octogenarians: The Lothian Birth Cohort 1936 study
Source: PLoS One. 2021 Jun 17;16(6):e0253153. doi: 10.1371/journal.pone.0253153 (PMC8211159; doi:10.1371/journal.pone.0253153)
Supplement: S4 Table — (DOCX) [file pone.0253153.s010.docx]

**S4 Table. LBC1936 participant responses to COVID-19 questionnaire: Social connectedness.**

| Social Connectedness questions | N | % |
| --- | --- | --- |
| Since the COVID-19 measures were introduced (23rd March 2020), has contact with your neighbours changed? |  |  |
| More contact now | 64 | 33.7 |
| The same | 89 | 46.8 |
| Less contact now | 37 | 19.4 |
| If contact with your neighbours has changed, how have you found this experience? |  |  |
| Positive | 63 | 62.4 |
| Neutral | 32 | 31.7 |
| Negative | 6 | 5.9 |
