# Supplementary material for: Impact of COVID-19 lockdown on psychosocial factors, health, and lifestyle in Scottish octogenarians: The Lothian Birth Cohort 1936 study
Source: PLoS One. 2021 Jun 17;16(6):e0253153. doi: 10.1371/journal.pone.0253153 (PMC8211159; doi:10.1371/journal.pone.0253153)
Supplement: S8 Table — (DOCX) [file pone.0253153.s014.docx]

S8 Table. Odds Ratios (95% Confidence Intervals) for decreased frequency of leaving the home since COVID-19 lockdown

|  | Model 1 | Model 2 | Model 3 | Model 4 |
| --- | --- | --- | --- | --- |
| Age^a^ | 0.941 (0.718 – 1.229) | 0.952 (0.726 – 1.245) | 0.904 (0.682 – 1.194) | 0.923 (0.692 – 1.229) |
| Sex Male | Reference | Reference | Reference | Reference |
| Female | 0.633 (0.368 – 1.087) | 0.598 (0.344 – 1.033) | 0.540 (0.303 – 0.955)* | 0.557 (0.303 – 1.018) |
| Adulthood occupational social class |  | 0.666 (0.495 – 0.893)** | 0.721 (0.524 – 0.989)* | 0.707 (0.508 – 0.978)* |
| General cognitive ability |  |  | 1.437 (1.041 – 1.990)* | 1.236 (0.878 – 1.743) |
| Number of chronic diseases |  |  |  | 0.872 (0.603 – 1.258) |
| Townsend disability scale score |  |  |  | 0.726 (0.459 – 1.130) |
| Self-reported health |  |  |  | 0.621 (0.415 – 0.920)* |

**p*<.05, ***p*<.01, ****p*<.001; Independent variables are from age-82 unless otherwise stated.

**^a^** Age is age in days at time of questionnaire (mean age 84).

Odds ratios for continuous variables based on 1SD change in independent variable.
