# Supplementary material for: Impact of COVID-19 lockdown on psychosocial factors, health, and lifestyle in Scottish octogenarians: The Lothian Birth Cohort 1936 study
Source: PLoS One. 2021 Jun 17;16(6):e0253153. doi: 10.1371/journal.pone.0253153 (PMC8211159; doi:10.1371/journal.pone.0253153)
Supplement: S9 Table — (DOCX) [file pone.0253153.s015.docx]

S9 Table. Odds Ratios (95% Confidence Intervals) for increased internet usage since COVID-19 lockdown

|  | Model 1 | Model 2 | Model 3 | Model 4 |
| --- | --- | --- | --- | --- |
| Age^a^ | 1.02 (0.75 – 1.40) | 1.003(0.730 -1.380) | 1.03(0.73 – 1.45) | 0.97 (0.68 – 1.38) |
| Sex Male | Reference | Reference | Reference | Reference |
| Female | 2.79( 1.50 – 5.30)** | 2.50(1.29 – 4.93)** | 2.34 (1.14 – 4.85)* | 2.32 (1.12 – 4.86)* |
| Living alone^b^ Alone |  | Reference | Reference | Reference |
| Not alone |  | 0.689(0.43 – 1.11) | 0.66 (0.39 – 1.10) | 0.65 (0.38 – 1.10) |
| General cognitive ability |  |  | 1.50 (1.02 -2.24)* | 1.53 (1.03 – 2.33)* |
| Anxiety symptoms |  |  |  | 1.31 (0.92 – 1.90) |

**p*<.05, ***p*<.01, ****p*<.001; Independent variables are from age-82 unless otherwise stated.

**^a^** Age is age in days at time of questionnaire (mean age 84).

**^b^** Living alone at time of questionnaire (mean age 84).

Odds ratios for continuous variables based on 1SD change in independent variable.
