# Supplementary material for: Impact of COVID-19 lockdown on psychosocial factors, health, and lifestyle in Scottish octogenarians: The Lothian Birth Cohort 1936 study
Source: PLoS One. 2021 Jun 17;16(6):e0253153. doi: 10.1371/journal.pone.0253153 (PMC8211159; doi:10.1371/journal.pone.0253153)
Supplement: S10 Table — (DOCX) [file pone.0253153.s016.docx]

S10 Table. Odds Ratios (95% Confidence Intervals) for reporting a greater change in daily routine since COVID-19 lockdown

|  | Model 1 | Model 2 | Model 3 |
| --- | --- | --- | --- |
| Age^a^ | 1.066 (0.818 – 1.392) | 1.125 (0.858 – 1.479) | 1.157 (0.857 – 1.567) |
| Sex Male | Reference | Reference | Reference |
| Female | 0.861 (0.498 – 1.485) | 1.170 (0.645 – 2.135) | 1.075 (0.547 – 2.124) |
| Living alone^b^ Alone |  | Reference | Reference |
| Not alone |  | 2.012 (1.098 – 3.727)* | 1.830 (0.920 – 3.683) |
| General Health Literacy score at mean age 73 |  |  | 0.687 (0.462 – 1.014) |

**p*<.05, ***p*<.01, ****p*<.001; Independent variables are from age-82 unless otherwise stated.

**^a^** Age is age in days at time of questionnaire (mean age 84).

**^b^** Living alone at time of questionnaire (mean age 84).

Odds ratios for continuous variables based on 1SD change in independent variable.
