# Supplementary material for: Impact of COVID-19 lockdown on psychosocial factors, health, and lifestyle in Scottish octogenarians: The Lothian Birth Cohort 1936 study
Source: PLoS One. 2021 Jun 17;16(6):e0253153. doi: 10.1371/journal.pone.0253153 (PMC8211159; doi:10.1371/journal.pone.0253153)
Supplement: S11 Table — (DOCX) [file pone.0253153.s017.docx]

S11 Table. Odds Ratios (95% Confidence Intervals) for reporting poorer self-reported physical health since COVID-19 lockdown measures introduced

|  | Model 1 | Model 2 | Model 3 | Model 4 | Model 5 |
| --- | --- | --- | --- | --- | --- |
| Age^a^ | 1.311 (0.976 – 1.767) | 1.379 (1.018 – 1.878)* | 1.502 (1.086 – 2.095)* | 1.469 (1.058 – 2.055)* | 1.450 (1.040 – 2.036)* |
| Sex Male | Reference | Reference | Reference | Reference | Reference |
| Female | 0.517 (0.287 – 0.925) | 0.544 (0.296 – 0.992)* | 0.522 (0.270 – 0.998) | 0.560 (0.285 – 1.088) | 0.559 (0.277 – 1.113) |
| General intelligence (g) |  | 0.589 (0.422 – 0.814)** | 0.772 (0.535 – 1.108) | 0.751 (0.517 – 1.083) | 0.725 (0.496 – 1.051) |
| Number of chronic diseases |  |  | 0.981 (0.661 – 1.456) | 0.942 (0.632 – 1.402) | 0.978 (0.651 – 1.467) |
| Townsend disability scale score |  |  | 1.480 (0.870 – 2.590) | 1.327 (0.763 – 2.350) | 1.311 (0.735 – 2.368) |
| Self-rated general health |  |  | 4.672 (2.821 – 7.985)*** | 4.492 (2.630 – 7.921)*** | 3.991 (2.310 – 7.110)*** |
| Anxiety symptoms |  |  |  | 0.983 (0.692 – 1.396) | 0.838 (0.536 – 1.301) |
| Depression symptoms |  |  |  | 1.306 (0.879 – 1.957) | 1.166 (0.766 – 1.783) |
| Emotional Stability |  |  |  |  | 0.807 (0.514 – 1.258) |
| Conscientiousness |  |  |  |  | 0.825 (0.566 – 1.198) |
| Extraversion |  |  |  |  | 0.827 (0.583 – 1.172) |

**p*<.05, ***p*<.01, ****p*<.001; Independent variables are from age-82 unless otherwise stated.

**^a^** Age is age in days at time of questionnaire (mean age 84).

Odds ratios for continuous variables based on 1SD change in independent variable.
