# Supplementary material for: Impact of COVID-19 lockdown on psychosocial factors, health, and lifestyle in Scottish octogenarians: The Lothian Birth Cohort 1936 study
Source: PLoS One. 2021 Jun 17;16(6):e0253153. doi: 10.1371/journal.pone.0253153 (PMC8211159; doi:10.1371/journal.pone.0253153)
Supplement: S12 Table — (DOCX) [file pone.0253153.s018.docx]

S12 Table. Odds Ratios (95% Confidence Intervals) for reporting poorer self-reported mental health since COVID-19 lockdown measures introduced

|  | Model 1 | Model 2 | Model 3 | Model 4 | Model 5 |
| --- | --- | --- | --- | --- | --- |
| Age^a^ | 1.343 (1.009 – 1.793)* | 1.308 (0.979 – 1.750) | 1.304 (0.972 – 1.755) | 1.273 (0.945 – 1.718) | 1.271 (0.939 – 1.726) |
| Sex Male | Reference | Reference | Reference | Reference | Reference |
| Female | 1.272 (0.729 – 2.227) | 1.044 (0.572 – 1.903) | 1.220 (0.648 – 2.299) | 1.098 (0.579 – 2.082) | 1.109 (0.570 – 2.159) |
| Living alone^b^  Alone |  | Reference | Reference | Reference | Reference |
| Not alone |  | 0.561 (0.307 – 1.017) | 0.582 (0.313 – 1.074) | 0.609 (0.325 – 1.132) | 0.533 (0.277 – 1.015) |
| Number of chronic diseases |  |  | 1.201 (0.836 – 1.730) | 1.113 (0.771 – 1.607) | 1.201 (0.824 – 1.754) |
| Self-rated general health |  |  | 2.015 (1.389 – 2.973)*** | 1.625 (1.099 – 2.434)* | 1.483 (0.992 – 2.238) |
| Anxiety symptoms* |  |  |  | 1.694 (1.213 – 2.381)** | 1.148 (0.760 – 1.732) |
| Depression symptoms* |  |  |  | 1.174 (0.832 – 1.664) | 1.033 (0.712 – 1.503) |
| Emotional Stability |  |  |  |  | 0.535 (0.351 – 0.806)** |
| Extraversion |  |  |  |  | 0.892 (0.641 – 1.242) |

**p*<.05, ***p*<.01, ****p*<.001; Independent variables are from age-82 unless otherwise stated.

**^a^** Age is age in days at time of questionnaire (mean age 84).

**^b^** Living alone at time of questionnaire (mean age 84).

Odds ratios for continuous variables based on 1SD change in independent variable.
