# Supplementary material for: Impact of COVID-19 lockdown on psychosocial factors, health, and lifestyle in Scottish octogenarians: The Lothian Birth Cohort 1936 study
Source: PLoS One. 2021 Jun 17;16(6):e0253153. doi: 10.1371/journal.pone.0253153 (PMC8211159; doi:10.1371/journal.pone.0253153)
Supplement: S13 Table — (DOCX) [file pone.0253153.s019.docx]

S13 table. Odds Ratios (95% Confidence Intervals) for experiencing COVID-19 related stress or nervousness during COVID-19 lockdown

|  | Model 1 | Model 2 | Model 3 | Model 4 |
| --- | --- | --- | --- | --- |
| Age^a^ | 0.98 (0.72 – 1.32) | 0.95 (0.70 – 1.29) | 0.92(0.66 – 1.29) | 0.94 (0.66 – 1.33) |
| Sex Male | Reference | Reference | Reference | Reference |
| Female | 1.71 (0.94 – 3.13) | 1.33 (0.69 – 2.53) | 1.24 (0.62 – 2.48) | 1.55 (0.73 – 3.31) |
| Living alone^b^  Alone |  | Reference | Reference | Reference |
| Not alone |  | 0.60 (0.38– 0.95)* | 0.62 (0.38 – 1.01) | 0.65 (0.38 – 1.11) |
| Anxiety symptoms |  |  | 1.75 (1.26 – 2.49)** | 0.99 (0.63 – 1.55) |
| Emotional stability |  |  |  | 0.40 (0.24 – 0.62)*** |

**p*<.05, ***p*<.01, ****p*<.001; Independent variables are from age-82 unless otherwise stated.

**^a^** Age is age in days at time of questionnaire (mean age 84).

**^b^** Living alone at time of questionnaire (mean age 84).

Odds ratios for continuous variables based on 1SD change in independent variable.
