# Supplementary material for: Impact of COVID-19 lockdown on psychosocial factors, health, and lifestyle in Scottish octogenarians: The Lothian Birth Cohort 1936 study
Source: PLoS One. 2021 Jun 17;16(6):e0253153. doi: 10.1371/journal.pone.0253153 (PMC8211159; doi:10.1371/journal.pone.0253153)
Supplement: S14 Table — (DOCX) [file pone.0253153.s020.docx]

S14 Table. Odds Ratios (95% Confidence Intervals) for experiencing loneliness during COVID-19 lockdown

|  | Model 1 | Model 2 | Model 3 | Model 4 | Model 5 |
| --- | --- | --- | --- | --- | --- |
| Age^a^ | 1.20 (0.999 – 1.006) | 1.13 (0.77 – 1.65) | 1.06 (0.71 – 1.61) | 1.06 (0.69 – 1.64) | 1.04 (0.68 – 1.62) |
| Sex Male | Reference | Reference | Reference | Reference | Reference |
| Female | 1.37 (0.71 – 2.84) | 0.66 (0.29 – 1.44) | 0.53 (0.21 – 1.27) | 0.41 (0.15 – 1.06) | 0.48 (0.17 – 1.26) |
| Living alone^b^  Alone |  | Reference | Reference | Reference | Reference |
| Not alone |  | 0.21 (0.11– 0.38)*** | 0.15 (0.07 – 0.30)*** | 0.14 (0.06 – 0.29)*** | 0.15 (0.07 – 0.31)*** |
| Townsend Disability Scale score |  |  | 1.64 (0.95– 2.90) | 1.79 (1.01 – 3.33) | 1.67 (0.92 – 3.14) |
| Self-rated general health Excellent |  |  | 0.10 (0.001 – 7.14) | 0.18 (0.001 – 26.09) | 0.17 (0.001 – 26.69) |
| Very good |  |  | 0.66 (0.01 – 32.39) | 0.76 (0.01 – 73.66) | 0.64 (0.01 – 65.46) |
| Good |  |  | 0.44 (0.01 – 21.26) | 0.42 (0.004 – 38.993) | 0.34 (0.003 – 33.48) |
| Fair |  |  | 5.48 (0.10 – 321.55) | 3.14 (0.03 – 356.73) | 3.32 (0.03 – 399.82) |
| Poor |  |  | Reference | Reference | Reference |
| Anxiety symptoms |  |  |  | 1.99 (1.26 – 3.27)** | 1.76 (1.01 – 3.14)* |
| Emotional stability |  |  |  |  | 0.76 (0.45 – 1.24) |

**p*<.05, ***p*<.01, ****p*<.001; Independent variables are from age-82 unless otherwise stated.

**^a^** Age is age in days at time of questionnaire (mean age 84).

**^b^** Living alone at time of questionnaire (mean age 84).

Odds ratios for continuous variables based on 1SD change in independent variable.
