# Supplementary material for: Impact of COVID-19 lockdown on psychosocial factors, health, and lifestyle in Scottish octogenarians: The Lothian Birth Cohort 1936 study
Source: PLoS One. 2021 Jun 17;16(6):e0253153. doi: 10.1371/journal.pone.0253153 (PMC8211159; doi:10.1371/journal.pone.0253153)
Supplement: S15 Table — (DOCX) [file pone.0253153.s021.docx]

S15 Table. Odds Ratios (95% Confidence Intervals) for a decrease in physical activity since COVID-19 lockdown measures introduced

|  | Model 1 | Model 2 | Model 3 |
| --- | --- | --- | --- |
| Age^a^ | 1.058 (0.807 – 1.389) | 1.054 (0.803 – 1.387) | 1.133 (0.854 – 1.506) |
| Sex Male | Reference | Reference | Reference |
| Female | 1.024 (0.601 – 1.748) | 1.133 (0.660 – 1.950) | 1.076 (0.614 – 1.891) |
| Adulthood occupational class |  | 1.586 (1.185 – 2.134)** | 1.426 (1.043 – 1.961)* |
| General cognitive ability (g) |  |  | 0.679 (0.491 – 0.931)* |

**p*<.05, ***p*<.01, ****p*<.001; Independent variables are from age-82 unless otherwise stated.

**^a^** Age is age in days at time of questionnaire (mean age 84).

Odds ratios for continuous variables based on 1SD change in independent variable.
