# Supplementary material for: Impact of COVID-19 lockdown on psychosocial factors, health, and lifestyle in Scottish octogenarians: The Lothian Birth Cohort 1936 study
Source: PLoS One. 2021 Jun 17;16(6):e0253153. doi: 10.1371/journal.pone.0253153 (PMC8211159; doi:10.1371/journal.pone.0253153)
Supplement: S16 Table — (DOCX) [file pone.0253153.s022.docx]

S16 Table. Odds Ratios (95% Confidence Intervals) for returning to or starting a new pastime since COVID-19 lockdown measures introduced

|  | Model 1 | Model 2 |
| --- | --- | --- |
| Age^a^ | 0.90 (0.67 – 1.22) | 0.92 (0.66 – 1.28) |
| Sex Male | Reference | Reference |
| Female | 2.02 (1.10 – 3.75)* | 1.89 (0.96 – 3.75) |
| General health literacy at age 73 |  | 1.36 (0.92– 2.02) |

**p*<.05, ***p*<.01, ****p*<.001; Independent variables are from age-82 unless otherwise stated.

**^a^** Age is age in days at time of questionnaire (mean age 84).

Odds ratios for continuous variables based on 1SD change in independent variable.
