# Supplementary material for: Impact of COVID-19 lockdown on psychosocial factors, health, and lifestyle in Scottish octogenarians: The Lothian Birth Cohort 1936 study
Source: PLoS One. 2021 Jun 17;16(6):e0253153. doi: 10.1371/journal.pone.0253153 (PMC8211159; doi:10.1371/journal.pone.0253153)
Supplement: Attachment — Submitted filename: Response to Reviewers.docx [file pone.0253153.s024.docx]

**Editor Comments**

**Please note: page and line numbers referenced in this document refer to the final edited ‘Manuscript.docx’ file, after all changes.**

**Comment 1.** Please ensure that your manuscript meets PLOS ONE's style requirements, including those for file naming. The PLOS ONE style templates can be found at

<https://journals.plos.org/plosone/s/file?id=wjVg/PLOSOne_formatting_sample_main_body.pdf> and

<https://journals.plos.org/plosone/s/file?id=ba62/PLOSOne_formatting_sample_title_authors_affiliations.pdf>

**Response:** Formatting of headings was updated to correct font size (Heading 1: 18pt, Heading 2: 16pt, Heading 3: 14pt) and italics in headings were removed. References to figures in the main text and tables in the supplementary materials were adjusted to reflect PLOS ONE’s naming conventions. All file names were also updated to reflect this naming convention, and supplementary tables S8-16, which had previously been grouped into one file, were separated into individually named files. Author affiliations were also updated to reflect guidelines relating to removal of building addresses and postcodes.

**Comment 2**. In the Methods section, please provide additional details regarding how the capacity to give informed consent was determined.

**Response:**  We note that all individuals invited to take part in the new wave of the LBC1936 study have participated in, or been in contact the LBC1936 study team, during five previous waves of testing. Indeed, a principle of the Adults with Incapacity Act is to take account of “the present and past wishes and feelings of the adult so far as they can be ascertained” (Section 1 (4) (a)) and this participation in multiple previous waves of the study could be understood as an indication of their past wishes and likely intent to continue participating. The following text has now been added to the Methods section (page 6-7, line 172-177):

*‘Instructions were included in the participant information sheet stating that for any participant requiring assistance with completing the online questionnaire, the person providing assistance should contact the LBC study team before beginning the questionnaire to confirm their status as a Welfare Power of Attorney, Guardian, or Nearest Relative. The LBC study medic specialising in geriatric medicine advised the team on whether this individual was suitable for providing consent on behalf of any participant lacking capacity to provide informed consent.’*

**Comment 3**. Please provide details regarding how the subset of participants from the Lothian Birth Cohort 1936 were selected for the current study. Please detail any inclusion and exclusion criterias applied.

**Response:** Details of the sampling method and inclusion criteria have now been added to the Methods section of our manuscript (page 6, line 168-170):

‘*The LBC1936 are a volunteer sample; inclusion criteria for the present study were defined as any participant who had completed at least one wave of testing, and who had the means to access and complete the questionnaire online by themselves or with assistance*.’

**Comment 4**. We note that you have indicated that data from this study are available upon request. PLOS only allows data to be available upon request if there are legal or ethical restrictions on sharing data publicly. For more information on unacceptable data access restrictions, please see <http://journals.plos.org/plosone/s/data-availability#loc-unacceptable-data-access-restrictions>.

In your revised cover letter, please address the following prompts:

a) If there are ethical or legal restrictions on sharing a de-identified data set, please explain them in detail (e.g., data contain potentially sensitive information, data are owned by a third-party organization, etc.) and who has imposed them (e.g., an ethics committee). Please also provide contact information for a data access committee, ethics committee, or other institutional body to which data requests may be sent.

b) If there are no restrictions, please upload the minimal anonymized data set necessary to replicate your study findings as either Supporting Information files or to a stable, public repository and provide us with the relevant URLs, DOIs, or accession numbers. For a list of acceptable repositories, please see <http://journals.plos.org/plosone/s/data-availability#loc-recommended-repositories>.

We will update your Data Availability statement on your behalf to reflect the information you provide.

**Response:** We have expanded on the process for accessing LBC1936 data, which contains sensitive and identifiable participant information, in our cover letter. For reference, this reads as follows**:**

***‘****We have also expanded on our data availability statement. Lothian Birth Cohort 1936 data contains sensitive, identifiable information about LBC1936 participants and consent was given only to provide data access to approved researchers. These data cannot be made public, but we are share data regularly with researchers nationally and internationally. This can be achieved by completed a data request form e-mailed to lbc1936@ed.ac.uk, and then via a formal Data Transfer Agreement between the University of Edinburgh and the researchers’ host institution. We have published full details of the data access procedure on our study website (*[*https://www.ed.ac.uk/lothian-birth-cohorts/data-access-collaboration*](https://www.ed.ac.uk/lothian-birth-cohorts/data-access-collaboration)*) alongside the requisite forms and data dictionaries. ‘*

**Comment 6.** Please include captions for *all* your Supporting Information files at the end of your manuscript, and update any in-text citations to match accordingly. Please see our Supporting Information guidelines for more information: [http://journals.plos.org/plosone/s/supporting-information](about:blank).Reviewers' comments:

**Response:** References to supplementary figures in the main text were adjusted to comply with naming conventions. Supplementary tables S8-16, which had previously been grouped into one file, were separated into individually named files. Individual captions for each supplementary file were added to the ‘Supporting information captions’ section (page 33-34, line 786-823).

**Reviewer #1**

**Thank you for the opportunity to review this manuscript. Authors examine the impact of COVID-19 on octogenarians. The manuscript is well presented and investigates the effects of the pandemic on an underrepresented population in the current researches. While the ms offers useful insights on this matter, there are some issues that need to be addressed.**

**Response:** We thank the Reviewer for their positive feedback and for their suggested changes which we hope will improve our manuscript.

**Comment 1.** Regarding the choice of covariates. Authors state that “Measures […] were selected a priori based on previous associations between these variables and psychosocial factors, health and lifestyle in the LBC1936 cohort”. Authors should elaborate on this point, ideally also referencing the appropriate literature.

**Response:** We have expanded on this point by referencing a number of papers which show associations between our covariate variables and psychosocial, health and lifestyle outcomes, mainly using LBC1936 data (page 11, line 199). The papers include the variables: childhood and adulthood socio-economic status, age, sex, education, marital status, living alone, area of residence, age-11 IQ, MMSE, gfluid, health literacy, comorbidities, lung function, grip strength, townsend, and BMI, extraversion, emotional stability, and conscientiousness.

**Comment 2.** What was the rationale behind choosing not to administer the anxiety/depression scale used in wave5 but only using the previous scores as a covariate? The issue I see here is that the comparison pre/during pandemic is only made on a self-report assessment based on a few questions. It would have been interesting to see the results of such comparison on a validated instrument: this could have offered a better estimate of the actual impact of the pandemic on the sample’s mental health.

**Response:** We agree that comparing anxiety and depression scores during lockdown with scores from the same scale measured previously would provide valuable information on changes in mood state over time. However, there were important logistical considerations which prevented us doing so. We were interested in examining a large range of outcomes with our LBC1936 COVID-19 survey while minimising burden on participants responding to the survey. As such, we prioritised scales for inclusion that i) best harmonised with other COVID-19 surveys in similar populations (e.g. Generation Scotland’s CovidLife survey) to allow for future multi-cohort analyses, and ii) were not prohibitively long. Anxiety and depression symptoms were previously measured in the LBC1936 study using the Hospital Anxiety and Depression Scale (Zigmond & Snaith, 1983) which itself consists of 14 questions. We judged that the benefits of including self-report questions used in the current survey outweighed that of including the longer HADS. However, we agree that it would have been optimal to include that were participant burden not considered, and have now added a mention of this point in the limitations section.

**Comment 3.** Discussion. Albeit, as suggested by Authors, studies in the general population may underrepresent octogenarians, a comparison with the existing literature could offer a more detailed interpretation of their results. To do so, a more thorough review of the literature is required. For instance, regarding adherence with protective measures, older aged groups are usually associated with higher compliance (e.g. Roma et al. 2020. How to improve compliance with protective health measures during the COVID-19 outbreak: testing a moderated mediation model and machine learning algorithms”. Int. J. Environ. Res. Public Health. <https://doi.org/10.3390/ijerph17197252>), and it would be interesting to have insight on what factors are associated with higher levels of compliance and how these findings relate to the literature regarding the general population. In other words, Authors should be more detailed in interpreting all of their results.

**Response:** The reviewer makes a good point here, and we have now expanded on the results regarding adherence to guidelines on leaving the home (page 23, line 432-437), and noted how this compares to some other recent papers about compliance to guidelines during the pandemic. Most notably, we describe how our results are in line with papers in the literature with regards to higher compliance in those with higher socio-economic status (Roma et al, 2020; Bish & Michie, 2010; Almutairi et al, 2020; Nivette et al, 2021), but contrasts with other papers which show associations between compliance and sex (Bish & Michie, 2010), and marital status (Almutairi et al, 2020). The manuscript now reads as follows:

*‘Unlike previous research on adherence to guidelines during the pandemic, we found no association between adherence to this guideline and sex (74) or marital status (75). Overall, results complement findings that the threat of COVID-19 is perceived to be lower for those who are healthier and have higher income (23), and is broadly in line with previous research showing associations between socio-economic factors such as level of education and pandemic guideline compliance (74–77).’*

**Comment 4.** Discussion pag.22 line 398. “associated with living alone and greater age-82 anxiety symptoms” there seems to be a mistake here.

**Response:** The text has been checked and is correct, but wording adjusted for clarity (page 22, line 417-418):

‘*Greater loneliness during lockdown was associated with living alone and greater age-82 anxiety symptoms*.’

**Comment 5**. Strengths and Limitations. Regarding the online formats and the consequent limitation of available respondents. For clarity, Authors should elaborate on this point, because it likely had a significant impact on their results.

**Response:** We agree that the use of an online surveys is a potential limitation which should be given careful consideration, particularly given the age demographic of the study population who may be less proficient and familiar with completing online surveys than younger individuals. Whereas the LBC1936 study typically administers paper-and-pencil type questionnaires, there were a number of logistical issues which informed our decision to use an online format for the LBC1936 COVID-19 survey. First, the study team were (and still are) working from home with no access to LBC studies’ offices to allow for preparation and receipt of physical questionnaires and the data entry required with written responses. Furthermore, it was important to administer the questionnaire during a limited time-period when the Scottish governments’ COVID-19 lockdown guidance was consistent across the entire Scottish population, to ensure that our results would not be confounded by differences in lockdown experience across the sample. An online format allowed us to overcome the issues related to working from home, and also allowed us to administer the survey and receive responses in a short time-period, before changes to the initial government lockdown guidance.

That said, we recognise that this might have led to some degree of bias, with our sample potentially more positively selected for individuals with home-access to the necessary equipment for online survey completion, and individuals with higher computer literacy. We also recognise that those who take part in studies and respond to surveys of this nature tend to represent those who score higher on many of our chosen covariate variables, often with more years of education, better general health, and higher scores on tests of cognitive ability. We demonstrate this in our comparison of responders and those who did not respond to the questionnaire (table 2 in the manuscript). That said, range restriction of this nature within the Lothian Birth Cohort has been shown to lead to modest underestimations of effects (see Deary et al., 2004; Gow et al., 2011; Johnson et al., 2012, 2010), so it is likely that results in this paper are, in fact, an underestimation of effects which might be found in a larger and less homogenous sample. We have expanded upon these points in the limitations section of our Discussion (page 24, line 471-483):

*‘The LBC1936 is a self-selecting sample consisting of mostly white Scottish participants who are likely to be healthier than the general population, and participants who have completed more waves of data collection have higher cognitive ability and physical fitness, and more professional occupational social class than those lost to attrition (44,45). Furthermore, as previously noted, responders to the LBC1936 COVID-19 questionnaire tended to have a more professional occupational status, more years of education, and better physical and cognitive ability than LBC1936 participants who did not respond. Consequently, the results reported here are likely to be modest underestimations of the true effect size in the general population, as has been demonstrated previously in this cohort (81–84).* *While reasons for lack of response are unknown, it is possible those who did not respond either did not have access to the necessary equipment to access the questionnaire online, or had lower computer literacy, further affecting generalisability of results. For example, if lower computer literacy is related to the outcomes under investigation in the current study, the results reported here are likely to be an underestimation of the true effect size in the population as a whole*.’

**Reviewer #2**

**Comment 1**. In methods criteria of inclusion of participant should be defined as well as type of sampling

**Response:** Details of the sampling method and inclusion criteria have now been added to the Methods section of our manuscript (page 6, line 168-170):

‘*The LBC1936 are a volunteer sample; inclusion criteria for the present study were defined as any participant who had completed at least one wave of testing, and who had the means to access and complete the questionnaire online by themselves or with assistance.*’

**Comment 2.** Please, I suggest to cite this papers on the relationship between psychosocial risk factors and physical health:

1) Chawla S, Kocher M. Physical activity at home during the COVID-19 lockdown in India: Need of the hour for optimum physical health and psychological resilience. J Health Soc Sci. 2020. 5(2): 187-192.

2) Chirico F, Heponiemi T, Pavlova M, Zaffina S, Magnavita N. Psychosocial Risk Prevention in a Global Occupational Health Perspective. A Descriptive Analysis. Int J Environ Res Public Health. 2019;16(14):2470. Published 2019 Jul 11. doi:10.3390/ijerph16142470

**Response:** We thank the reviewer for these reference recommendations, which we have now included in the Introduction (page 3-4, line 85-87). We also added a reference to a paper recently published by our team members in the LBC1936 study on physical activity and psychosocial risk factors (page 4, line 87-90). The text now reads as follows:

*‘Prior research indicates the relationship between physical health and psychosocial risk factors is relevant for the COVID-19 pandemic (19,20). Recent research examining the same data as in the current study found that levels of physical activity significantly decline during lockdown, and higher levels of physical activity during lockdown was associated with greater extraversion and a higher age 11 IQ (21).’*

**Reviewer #3**

**Summary:** This is a cross-sectional survey conducted in a subset of Lothian Birth Cohort (LBC) 1936 Study, aiming to examine the effects of the Scottish COVID-19 lockdown on psychosocial factors, health, and lifestyle among Scottish octogenarians. A total of 190 participants with a mean age of 84 years, responded to the online questionnaire survey in May 2020. The authors concluded that individual cognitive function, occupational class, self-rated health, anxiety, emotional stability, and living alone may be related to risk of lockdown-related psychosocial and physical outcomes. The manuscript is overall well-written.

**Strengths:**
1. A unique population: to date, there is limited COVID-19 literature on the impact of lock down on older adults in community-dwelling. This study offers a snapshot of two months lock down on Scottish octogenarians.
2. Leveraging the Lothian Birth Cohort: Its key advantage is that it has a well-defined population with rich data on cognitive ability, demographics, psychosocial, and health factors previously collected at age 73 (2007-2010, n=866), 76 (2011-2013, n=697), 79 (2014-2017, n=550), and 82 (2017-2019, n=431).

**Response:** We thank the Reviewer for their positive feedback, recognising important strengths of our study.

**Comment 1.** It is helpful to include S8 table as a main table so the readers can have a good picture of the study participants characteristics. It would be helpful to include total sample size for respondents and non-respondents.

**Response:** We thank the Reviewer for this suggestion. Table S8 has now been added to the manuscript as table 2 (page 12, line 217), and details of the total sample size for respondents and non-respondents has been added to the table.

**Comment 2.** How representative of this subsample as compared the Scottish older people in general?

**Response:** Selectivity bias due to volunteer sampling and attrition across waves of data collection is a limitation in the majority of longitudinal observational cohort studies. As noted in our discussion, and reported previously, participants from LBC1936 have higher socioeconomic status and cognitive ability compared to the general population, and are likely to also have higher physical fitness. We have expanded upon the implications on this in the limitations section of our Discussion (page 24, line 471-478):

*‘The LBC1936 is a self-selecting sample consisting of mostly white Scottish participants who are likely to be healthier than the general population, and participants who have completed more waves of data collection have higher cognitive ability and physical fitness, and more professional occupational social class than those lost to attrition (44,45). Furthermore, as previously noted, responders to the LBC1936 COVID-19 questionnaire tended to have a more professional occupational status, more years of education, and better physical and cognitive ability than LBC1936 participants who did not respond. Consequently, the results reported here are likely to be modest underestimations of the true effect size in the general population, as has been demonstrated previously in this cohort (81–84).’*

**Comment 3.** How to separate natural physical and cognitive decline due to aging from COVID-19 impact?

**Response**: We thank the reviewer for raising this important point. This is a consideration for any analysis using LBC1936 data, given the study is longitudinal in nature, so any changes over time may reflect the influence of specific events, in this case, the COVID-19 lockdown, but outcomes may also be affected by factors outwith our control. Our outcomes in this case can be directly linked with the COVID-19 pandemic, either because they are specific to the pandemic itself (for example, adherence to ‘leaving the home’ guidance), or because we have measured these same outcomes before and after the onset of lockdown (for example, asking participants to rate their health before and after lockdown). We can therefore be confident that significant effects found are due to the effect of COVID-19 lockdown, rather than reflecting changes which would have happened regardless between wave 5 data collection (participant mean age 82) and data collection during the time of the COVID-19 pandemic (participant mean age 84).

Whereas it is not possible to entirely separate the effects of the COVID-19 pandemic from natural ageing-related effects, evidence supporting the position that our results are not solely a consequence of ageing comes from a recent publication (Okely et al, 2020, <https://doi.org/10.3390/ijerph18010210>) based on the same cohort examined in the current paper. Okely and colleagues tested whether the effect of ageing on social support and physical activity measured before the COVID-19 pandemic (between age 79 and 82) was of comparable magnitude to changes in these factors reported by LBC1936 participants during lockdown (between ages 82 and 84). Whereas small significant changes in social support and physical activity were found during lockdown, changes in these factors between age 79 and 82 were not significant. As such, it is likely that the results reported here are due to the influence of the COVID-19 pandemic rather than being a result of general ageing.

Upon reflection, we have amended the limitations section of our Discussion to acknowledge this issue (page 25-26, line 492-500):

*‘In addition, though we have interpreted results as being a consequence of the COVID-19 lockdown and associated guidance, participants were, on average, 2 years older than they were at their most recent assessment, therefore we cannot rule out the possibility that results were somewhat influenced by age-related physical and cognitive declines. Recently published analyses based on the same cohort as the present study have demonstrated that while there were small but significant changes in social support and physical activity variables between wave 5 (age 82) and the beginning of the COVID-19 pandemic (age 84), changes in social support and physical activity prior to the pandemic (between age 79 and 82), assumed to reflect ageing-related effects, were not significant (21). As such, it is likely that results reported in the current study were not due solely to age-related change.’*

**Reviewer #4**

Great opportunity to understand the impact of COVID-19 on this older population. I think this is an important contribution to the literature but some work needs to help the readers easily digest the material.

Overall, I think the authors need to be a bit more careful in their statements. I think the intention vs the actual words may not match perfectly. I’ve noted places where I was concerned about the statement being bolder than the authors meant it to be. There was too much vagueness on ‘older’ population and how this was defined. Many statement included the word older adults and that could be anyone age 50 and older…but since it was not specified, super unclear. The methods section needs to be more clearly presented as does the results section.

**Response:** We are grateful to the Reviewer for their positive and encouraging feedback, and welcome their suggested amendments which we have implemented, to the benefit of our manuscript.

**Comment 1.** Abstract, please add place (I’m sorry I do not know where Lothian Birth Cohort participants live).

**Response:** The geographical location of LBC1936 participants has now been added to the abstract (page 2, line 33-34).

‘*Lothian Birth Cohort 1936 members, residing mostly in Edinburgh and the surrounding Lothians regions in Scotland*…’

**Comment 2.** “The physical, psychological, and social effects of coronavirus disease 2019 (COVID-19) are unprecedented.” Line 51
This is a bit of an overstatement. Historically, there were other epidemics/pandemics that had incredible impact, we just did not have the scientific and communication systems in place to document as well. This is unprecedented in our lifetime only and has impacted developed countries.

**Response:** We thank the Reviewer for this valid comment, and have removed this section of text (page 3, line 58).

**Comment 3.** “We are yet to discover the effects of COVID-19 lockdown measures, particularly on older people” line 56 There is actually papers out that discuss the effect; it all depends on how ‘older people’ are defined. I suggest you make this more specific—particularly on people over 80 years of age and this when then be more accurate.

**Response:** We have amended the text to clarify that effects of COVID-19 lockdown measures, specifically in the over 80s age group, are yet to be fully determined (page 3, line 62-63):

*‘The effects of COVID-19 lockdown measures on older people are yet to be determined, especially in those over the age of 80, who are classed as a ‘high risk’ group in Scotland (3).’*

**Comment 4.** “have therefore endured some of the greatest restrictions for the 58 longest period.” Line 57. I would suggest placing this in context of UK? Or Scotland? This has not been true for all counties.

**Response:** We have amended the text to clarify that this refers to older adults in Scotland (page 3, line 62-63):

‘*The effects of COVID-19 lockdown measures on older people are yet to be determined, especially in those over the age of 80, who are classed as a ‘high risk’ group in Scotland (3).’*

**Comment 5.** ”at highest risk of severe illness” line 62. In the highest risk of severe illness category, right? Not the highest risk and your citation is on mortality which one could argue is severe illness but often this is being used to mean ICU admission, ECMO, ventilation… Either different citation or change to mortality and adjust the statement accordingly.

**Response:** We are grateful to the Reviewer for bringing this to our attention. The text and order of citations has been amended in this entire paragraph to make the distinction between illness outcomes and mortality clearer (page 3, line 68-77):

*‘In Scotland, older people are considered to be at higher risk of severe illness from COVID-19 (3–6). he risk increases for individuals with chronic comorbidities, particularly ageing-related diseases including cardiovascular disease, diabetes, respiratory and chronic pulmonary disease (5,6). Over half of those aged over 80 are estimated to be at high risk due to underlying health conditions (7). Older people are also at higher risk of mortality; in Scotland, 77% of all deaths involving COVID-19 to 14th June 2020 were of people aged 75 and over (8). A prospective cohort study of individuals aged between 0 and 104 (median age 73) in UK acute care hospitals found the highest proportion of hospitalisations and mortality among those aged 80 and over (5). Because of their increased risk, those in the ‘high risk’ category when lockdown began in Scotland were asked to ‘shield’, remaining at home and strictly avoiding social contact with anyone outside of their homes for at least 12 weeks.’*

**Comment 6.** A prospective cohort study in UK acute care hospitals found the highest proportion of hospitalisations and mortality among those aged 80 and over (4). (line 64). What was the comparison group? I suspect other ages, right? Not those with HIV or multiple comorbidities or…? Need to add the comparison group.

**Response:** Details of the comparison group have now been added to the text (page 3, line 73-75):

‘*A prospective cohort study of individuals aged between 0 and 104 (median age 73) in UK acute care hospitals found the highest proportion of hospitalisations and mortality among those aged 80 and over (5).*’

**Comment 7.** There is a bit of a mishmash between severity (hospitalization) vs mortality in the intro. It would be cleaner to put the concepts together more logically.

**Response:** Please see response to question 5, which we hope also addresses this comment.

**Comment 8.** “Because of this increased risk, those most vulnerable to the virus when lockdown began were asked to ‘shield’, remaining at home and strictly avoiding social contact with anyone outside
of their homes for at least 12 weeks.” Line 70. Again need place since this is a global sentence.

**Response:** We have amended the text to clarify that this refers to older adults in Scotland (page 3, line 75-77):

‘*Because of their increased risk, those in the ‘high risk’ category when lockdown began in Scotland were asked to ‘shield’, remaining at home and strictly avoiding social contact with anyone outside of their homes for at least 12 weeks.*’

**Comment 9.** As you use vulnerable to describe at risk population, please define vulnerable, particularly the age at which someone is considered vulnerable. All you say is older people which I do not know what that means precisely. Similarly, the use of ‘older people’ is just vague and needs clarity. I would suggest defining it to fit with your study. “Data from older people during the pandemic are surprisingly limited. Studies of this age group are underrepresented in COVID-19 literature to date, “ (line 85) This entirely begs the question of ‘this age group”

**Response:** We agree that the term vulnerable is somewhat vague. We have removed this phrase and specified that older adults over the age of 80 are particularly underrepresented in the literature to date (page 4, line 95-96):

*‘Data from older people during the pandemic are limited. The current study is particularly concerned with those over the age of 80, who are under-represented in the COVID-19 literature to date.’*

**Comment 10.** ”Data from older people during the pandemic are surprisingly limited.”line 85 Why is this surprising? Very few surveys have sufficient numbers of people in this age range.

**Response:** We thank the reviewer for this comment. Given that older people over the age of 80 are most at risk from COVID-19, and have therefore had to adhere to more stringent measures during the pandemic, we believe that, despite some of the challenges which might be associated with data collection in older participants, it is surprising that fewer researchers have attempted to collect data on this demographic. We have therefore clarified our use of the word ‘surprisingly’ in the text (page 4, line 96-97), which now reads:

*‘Data from older people during the pandemic are surprisingly limited, given that they are ‘high risk’ and any effects of strict lockdown in this group are not clear.’*

.

**Comment 11.** “In studies which do include older adults, they often account for only 2-27% of the overall sample (19–22), with results based on fewer than 50 older adults in some cases” line 90 This does not support your argument that there are limited number of participants in a study if ¼ are older and ‘some case” but presumably not all have less than 50. Need to rephrase this whole argument. Recommend you keep it focused on your contribution of over 80 year olds. That is good enough. You don’t need a ton to justify this study.

**Response:** We thank the reviewer for their suggestion and have slightly rephrased this section (page 4, line 96-102):

*‘Data from older people during the pandemic are surprisingly limited, given that they are ‘high risk’ and any effects of strict lockdown in this group are not clear. The current study is particularly concerned with those over the age of 80, who are under-represented in the COVID-19 literature. No study to date has focused solely on adults over the age of 80. Even in high-quality large-scale studies of COVID-19 with representative samples of hundreds or thousands of participants, the number of individuals sampled over the age of 80 is low (23–25), with conclusions drawn from sample sizes fewer than as low as 20 (26), and in some studies those over 80 are excluded entirely (27).’*

**Comment 12.** There is also a fair amount of jumping between UK and Scotland. Why say UK lockdown since your population, I presume was Scotland. Lockdown in Scotland?

**Response:** We agree that in the context of our study, discussion of lockdown in Scotland is sufficient. Any references to UK lockdown have been removed.

**Comment 13.** Please provide url for reports since the citations don’t actually work to get to the reports.

Individuals aged over 75 were more than twice as likely to report high anxiety during lockdown compared to those under 24 (31). Line 98. Why the comparison with very young adults? Kind of jarring. I looked up this report and I couldn’t find the information so don’t know who it was reported (such as by age group).

**Response:** The URL is provided in the References section (reference 35). This URL has been checked and is working:

[www.ons.gov.uk/peoplepopulationandcommunity/wellbeing/articles/coronavirusandanxietygreatbritain/3april2020to10may2020](http://www.ons.gov.uk/peoplepopulationandcommunity/wellbeing/articles/coronavirusandanxietygreatbritain/3april2020to10may2020)

The information about anxiety in older adults compared to other age groups can be found under the ‘Age’ subsection of Section 6 ‘Personal characteristics associated with high anxiety’. We have amended the text in our manuscript to reflect that individuals over the age of 75 have higher anxiety than any other age group, not just young adults (page 4, line 109):

‘*Individuals aged over 75 were more likely to report high anxiety than any other age group (32*).’

**Comment 14.** mostly middle aged and older adults” line 101 please define, age xxx

**Response:** The text has now been amended to give age ranges (page 4-5, line 112-117):

‘*(29). One of few studies to report on mostly middle aged and older adults (aged 23-88, with 63% of the sample over age 60) found differences in COVID-19 knowledge, awareness, attitudes, and behaviours across ethnic and socioeconomic groups, and in relation to differing levels of health literacy (23); being unemployed or retired, having poorer health, and having lower health literacy were associated with poorer COVID-19 knowledge and fewer changes to daily routine.’*

**Comment 15.** To date, they have attended four further
152 waves at mean ages 73 (2007-2010, n=866), 76 (2011-2013, n=697), 79 (2014-2017, n=550), and 82
153 (2017-2019, n=431). Probably not necessary to provide mean age; more important is why there is apprx 50% retention—dying or unable to participant to addresses the potential bias of these data. And 25% of these completing the survey. . For example does this sample basically represent ‘healthy 80+ year olds with high computer literacy? It is OK, but maybe a limitation of the study and I would suspect the results would be even stronger with less healthy folks included but we just don’t know

**Response:** As described in our recent Cohort Profile Update paper (reference 45), the main reasons for attrition in the LBC1936 study are death and illness, both are unavoidable causes of in longitudinal cohort studies of older adult populations. We not that the 20% attrition rate across each 3-year period compares favourably with other similar longitudinal studies (though they are across wider age ranges and younger participants). Participants who remain in the study are known to have higher cognitive ability and physical fitness, and more professional occupational social class. Additionally, because the questionnaire was administered in an online format, the current sample might also have higher computer literacy. As such, and as the Reviewer suggests, it is possible that the effects reported here are underestimates. This is an important point to address and as such we have expanded on this in the limitations section of our Discussion (page 24, line 471-483):

‘*The LBC1936 is a self-selecting sample consisting of mostly white Scottish participants who are likely to be healthier than the general population, and participants who have completed more waves of data collection have higher cognitive ability and physical fitness, and more professional occupational social class than those lost to attrition (44,45). Furthermore, as previously noted, responders to the LBC1936 COVID-19 questionnaire tended to have a more professional occupational status, more years of education, and better physical and cognitive ability than LBC1936 participants who did not respond. Consequently, the results reported here are likely to be modest underestimations of the true effect size in the general population, as has been demonstrated previously in this cohort (81–84). While reasons for lack of response are unknown, it is possible those who did not respond either did not have access to the necessary equipment to access the questionnaire online, or had lower computer literacy, further affecting generalisability of results. For example, if lower computer literacy is related to the outcomes under investigation in the current study, the results reported here are likely to be an underestimation of the true effect size in the population as a whole.’*

**Comment 16.** I’m not a fan of having the questionnaire listed in supplemental. No indication of validity or reliability of the questions; much too vague to me; as this is online, not sure why this isn’t included in some detail in the measure section. It seems like the questions were included in the table but it is not clear. What was each instrument? Most of these seem very genera and beg the question of validity without it being specified. A table that lists the different instruments and their validity/reliability would strengthen the paper; or if these have not been tested, then citations that used these questions.

**Response:** We have amended the text in the ‘LBC1936 COVID-19 questionnaire’ section (page 6-7, line 165-186) to aid the reader in understanding the contents of the questionnaire. However, due to the length of the questionnaire we do not think it is appropriate to include the full questionnaire in the measures section.

Where widely available standardised scales have been used, references for these scales are given in Table 1 (page 8-10). These scales have all been used in previous waves of the LBC1936 study. Where bespoke questions have been used (the majority of which were specific to COVID-19 lockdown guidance), the design of the questions was informed by and chosen to harmonise with other active, publicly available COVID-19 surveys (e.g. Generation Scotland’s CovidLife survey and the Chicago COVID-19 Comorbidities (C3) Survey). We have edited the manuscript to make this clearer (Page 7, line 181-185):

*‘Questions were designed to harmonise with existing COVID-19 surveys including Generation Scotland’s CovidLife Survey (46), the Chicago COVID-19 Comorbidities (C3) Survey (47), and surveys from other studies of COVID-19 attitudes and behaviours (23). Many questions were adapted from these surveys and had Likert-type response scales (23,46); all were optional.’*

**Comment 17**. It seems that sometime in May surveys were sent and potentially completed. The survey questions ask about the previous 6-10 weeks, last week in March, all of April, and then sometime in May. Most of the questions assume either average over this period or more likely people will respond to their behavior in the past week or two. This is a limitation to the study since there could very well be a lack of consistency by respondents in thinking about this time period as well as answering it accurately for this time period.

**Response:** We acknowledge the concern of the reviewer, however we feel that best efforts were made in the wording of individual questionnaire items to control for variability in the time period on which participants based their responses. It is important to note that during the time that our questionnaire was live, the Scottish government lockdown guidance did not change, and was the same for all individuals in Scotland. Therefore, even if some participants gave answers in relation to the past week rather than the entire lockdown period, we can be certain that responses were not confounded by variations in lockdown guidance. We agree that this concern should be addressed in the manuscript, and as such, have added the following text to the ‘Strengths and Limitations’ section (page 24-25, line 483-491):

*‘It could also be considered a limitation that COVID-19 survey questions referred to a relatively long period of time from lockdown to survey completion, spanning 2 months, therefore responses may lack consistency if some participants respond while only reflecting on their most recent behaviour and experiences. However, this is likely to have been substantially mitigated by the fact that questions were explicitly worded in order to control for any variability in the period on which participants based their answers. In addition, Scottish Government guidance for those over age 80 did not change at any point while the LBC1936 COVID-19 questionnaire was live, therefore it in not possible that responses would have been confounded by any change in lockdown guidance.’*

**Comment 18**. Collected at wave 1 (mean age 70). For sex row; age not necessary.
Question collected previously need to have more clear description. Long list of cognitive ability and just using citations leave much work to the readers to sort this out.

**Response:** The decision to include the wave and age of data collection for the sex variable in Table 1 was for consistency with the overall format of the table; this information has been provided for all variables previously measured in the LB1936 study and included in the current manuscript.

We have added more detail to the cognitive ability measures described in Table 1 (page 9).

**Comment 19.** Was BMI components measured or self-reported?

**Response:** The BMI variable was based on height and weight measurements obtained by a trained research nurse. We have expanded upon the description of this variable in table 1 (page 9).

**Comment 20.** The table was very difficult to process. It would be easier if broken up between the 3 purposes (*, a, b)

**Response:** We agree that the table notes previously included were confusing to follow, and on reflection some were unnecessary. The table notes for table 1 (page 10) have been amended, which we hope improves the readability of the table.

**Comment 21.** “10 COVID-19 questionnaire outcomes” line 206. What is this. Super difficult to follow. Of these correlated variables, were they all considered to be stable and not have any meaningful change from time of collection to current?

**Response:** We agree that the wording of this sentence made it difficult to follow, and as such the text has now been amended (page 15, line 227-230):

*‘We conducted exploratory bivariate Spearman’s rank correlations to identify relationships between previously measured characteristics and COVID-19 questionnaire outcomes from the subthemes: adherence to guidance, impact on day-to-day living, self-reported physical and mental health and loneliness, and lifestyle.’*

The majority of previously measured characteristics (i.e., demographics, health, and personality) are expected to have showed minimal change between wave 5 data collection at age 82 and during the COVID-19 pandemic at age 84. For example, between 2017 and 2021, proportions of chronic disease in the LBC1936 have remained relatively stable (no change in hypertension (~57%) or diabetes (~12%) and only a small increase from 37% to 39.4% in cardiovascular disease); and individual differences in personality factors are known to be relatively stable (see Mõttus, Johnson & Deary, 2012, doi: 10.1037/a0023690). Although some ageing-related decline in some cognitive ability factors might be expected over this time period, due to the nature of the online questionnaire, it was not possible to re-assess participants’ cognitive ability during the pandemic. Furthermore, some have argued that two waves of longitudinal data collection over a short period such as the one examined in the current paper are not sufficient to examine meaningful ageing-related change (see Johnson et al., 2012: <http://dx.doi.org/10.14301/llcs.v3i3.198>).

**Comment 22.** “grouped into blocks by variable type” no idea what this means

**Response:** We apologise for this description being unclear and have now amended the text (page 14, line 233-236):

‘*Additional covariates for each model were selected on the basis of significant correlations with COVID-19 questionnaire outcomes. These were entered consecutively into regression models based on variable subtype in the following order: age and sex, demographics, cognitive ability, health, mood, personality.’*

**Comment 23.** “between ‘before’ and ‘during’ lockdown ratings for self-reported physical and mental health” line 220 not clear what questions were used for this? Validated questions or not?

**Response:** These are adaptations of questions included in the well-validated Short Form 36 (SF-36). The self-reported physical health question from this questionnaire has previously been administered in the LBC1936 study, and the mental health question was based on a questionnaire included in Generation Scotland’s CovidLife survey. Additional details have been added to the measurement description in table 1 (page 8-10).

**Comment 24.** The long correlation between age 82 and 84 was way too much text; a table is sufficient; similarly, there is considerable redundancy between the text and table 3. Both are unnecessary. If the text had less numbers in it, it would read easier, Lump those that go in the same direction and refer to the table for specific odds.

**Response:** We agree that there was substantial redundancy between the tables and text. Correlation coefficients and odds ratios have now been removed from the text, and we have slightly amended the format of the text to improve readability (page 17 and 19, line 321-353; page 19 and 21, line 358-384).

**Comment 25.** To me what was missing was looking at gender. So stratifying by gender since the risk for severe COVID infection was so much higher for men vs women; did gender influence the outcomes. I would also imagine living alone and male vs female would also be a very different story w/r to impact of COVID but don’t know if you are sufficiently powered.

**Response:** Associations between COVID-19 related outcomes and sex were examined in correlation analyses (Table 3, page 20). In this sample, female sex was correlated with using the internet more often, better self-reported physical health and taking up a new pastime or returning to an old one during lockdown. Due to the limited associations between sex and outcomes of interest in the current sample, and relatively small samples sizes, we agree with the reviewer’s point about statistical power.

**Comment 26.**  On octogenarians” line 384. Is this true, they were all in their 80s? If so that is a great way to describe this population from the beginning. This was not obvious until the discussion and it should have been part of the intro…

**Response:** We attempted to be clear from the outset that all members of this cohort were born in 1936 with a mean age of 84 at the time of LBC1936 COVID-19 questionnaire completion. We provided this information in the Abstract (page 2, line 34), Introduction (page 3, line 65 and page 5, line 127-130), and Methods (page 6, line 149 and 157).

**Comment 27.** “which may be underestimates of the experiences in the general population.” Line 447. This will never generalize to the general population; but it could generalize to octogenarians who face lock down but more likely generalizes to this study since there were so many significant differences between those who completed this study and those who did not, as noted in the results section.

**Response:** We agree that our results may not be fully generalizable and have expanded upon this point in the limitations section of our discussion (please see our response to comment 15).
